# University of New-York—Medical Department—Annual Announcement of Lectures—Session 1848-9

**Published:** 1848-06

**Authors:** 


### EDITORIAL DEPARTMENT.

*University of New-York—Medical Department—Annual Announcement of Lectures—Session* 1848–9.

Our readers are, of course, aware that the National Medical Convention of 1847, recommended the extension of the lecture terms, in Medical Colleges, from four, to six months. At the last meeting of the Association, as we learn, this subject was reconsidered, and it was concluded that, for the present, it was advisable to recommend an extension to five months only. In the mean time, several Colleges had adopted the change so far as to extend their terms to five months.

The University of the City of New-York, in its circular recently issued for the session of 1848–9, takes ground against the propriety of extending the lecture term. We quote the following remarks on that subject:—

" An opinion has obtained in many parts of the country in favor of an increased period of College instruction, and several attempts have been made to bring about that result. In some institutions the number of daily lectures has been reduced, and the time spent in college attendance correspondingly increased. But in this arrangement there are serious evils. Students who come from a distance to attend medical colleges, come with the expectation of devoting their time exclusively to that pursuit. In the majority of cases they are strangers in the cities to which they are temporarily brought. If, after three or four morning hours, spent in the lecture room, they have no further employment for the rest of the day, their time is unduly wasted, and they are put to a needless expense. It is sometimes said that an attendance on six lectures a day is inconsistent with health. Experience shows how illusory the idea is. In almost all colleges not only do the young men attend the prescribed courses, but a majority of them, in addition, voluntarily avail themselves of the private instructions of resident physicians, who receive classes for lectures or examinations. It ought also to be remembered that there is no obligation to graduate at the end of the second course. In this Institution, as in most others, all subsequent courses are without charge, and the student is free to expend as much time in the acquisition of information as he is disposed. Moreover, there is another point of view in which the system of reducing the number of lectures given each day and increasing the time spent in the cities, ought to be regarded by those having an interest in education. To have his time fully occupied is one of the great preservatives of a student from those temptations in which all cities abound. It requires no sagacity to foresee what innumerable evils surround him if he is dismissed with half the day upon his hands. The system of spreading the usual amount of instruction over a long period, is not even an apparent improvement; it is a positive evil; and, whether we regard it as respects expense, or health, or morals, it is better that a student should attend six lectures a day for four months than four lectures a day for six months."

The arguments of the University in favor of the position it has taken, as the reader will perceive, are based on the supposition that if the lecture term be extended, the number of lectures on each day are necessarily to be reduced. It is true, it has been generally stated as a reason why the term should be extended, that the number of lectures now usually given daily (six) are too many; but it does not follow as a matter of course, that the number of lectures shall be diminished, provided the terms be extended. If it be the deliberate opinion of the University of New-York, that six lectures per day are not too many, they might still extend the term, and continue to have the same number as at present. Conceding all that the University assumes, it will not be denied that a five month's course, of six lectures per day, is worth more than a four month's course; nor will it be contended that each of the departments of medical instruction does not admit, with advantage to the student, of a five month's course, notwithstanding six lectures may be given daily.

The opinion, however, is very generally entertained, as already remarked, that six lectures a day are more than the student can profitably attend to; that the mental ingesta is too great and varied, for healthy digestion to take place. The Faculty of the University of the city of New-York, we suspect, are alone in the position they have taken. Let us briefly examine the considerations which they advance to sustain their position. The first objection to diminishing the number of daily lectures is, that students, coming from a distance, expect to have their time fully occupied with lectures, and, unless they hear, at least, six daily, feel that they do not receive a fair equivalent for their time and money. It might be said in answer to this objection, that the matter is one which should not be left to the student's judgment. It is a matter concerning which the student cannot be expected to judge so correctly as members of the profession, and especially those who have directed their attention to medical education. We do not say that the experience of students is not to be consulted, but that it does not fall within their legislative province. Medical institutions do not consult the wishes of students in other matters, such as the requirements for graduation; why appeal to them in this matter? But, waiving this, is it true that students prefer six daily lectures to a lesser number? So far as our own inquiries go, (and we have devoted some attention to this point) students would much prefer a lesser number. They are sensible that they cannot reap the advantages from six lectures daily, which they might do from the same number of lectures now constituting a course, distributed over a longer period. The attention becomes fatigued; the succession of so many subjects, following so closely upon each other, involves confusion, and over-tasks the memory; and they have neither time nor strength for reflection, reading and catechetical exercises, in connection with the lectures. Besides many depend upon the opportunities afforded at the lecture term for dissections, and there are to be added to them other duties. We believe if the question were to be submitted to any class, whether they would hear four or six lectures daily, they would decide upon the former.

The Faculty of the University of the city of New-York, consider the idea that attendance upon six lectures daily is prejudicial to health, to be illusory, We doubt, however, if experience will bear out this assertion. That many are able to endure it, is not evidence that it does not compromise the health in not a few instances. Students often, if not generally, will prefer to sacrifice lectures, rather than sacrifice health, and, accordingly, if their powers of endurance fail, they forego regular attendance upon some branches. But we suspect the history of every lecture term will disclose instances, more or less in number, in which students have felt compelled to leave before the end of the term, on account of impaired health; and that many suffer from application and confinement to the lecture room, is a matter of common observation. If this be not the case at the University of the city New-York, we should incline to the opinion that the students at that Institution are more careful not to compromise health by constant application and attendance, than at other Colleges.

The objection upon which most stress is laid, appears to us the most specious of all. It is, that unless the student is incessantly occupied, he is liable to yield to the temptations surrounding him, and fall into evil indulgences. It is to be hoped that this conveys an unjust reflection upon the character of medical students generally; but, assuredly, they who are not proof against city temptations, will not be restrained by the fact that lectures are given, attendance upon all of which is, by no means, obligatory. Besides, admitting that one or two hours are in this way provided for, where is the security for the eighteen hours still remaining. If students have no better protection against evil indulgences than is afforded by the privilege of attending six lectures daily, their situation it is to be feared, is irremediable! If the experience of the schools in New-York furnish data for such a conclusion, we think our readers will at once agree with us, that it is far better to attend lectures at places where the temptations to evil doing are fewer than in that city. There is doubtless some force in this consideration. A city of forty or fifty thousand inhabitants supplies, abundantly, material for clinical study, and is far less obnoxious to the dangers of very large cities, like the city of New-York! Individually we have no objection to conceding to the last mentioned objection all the facts upon which it is presumed to be based.

But the medical announcement from which we have quoted, virtually refutes its own positions. Directly following the extract already given, is this paragraph:—"But there is a way in which a real improvement can be effected. It is the plan which has been for years adopted by this Faculty; to leave the long established winter course, with its six daily lectures, untouched, and give, *in addition*, a series of lectures in the month of October *without charge*. To the advantages of this plan the attention of physicians is asked. Commencing with the first Monday in October, and continuing until the introductories of the winter course, lectures are given each day in this Institution. No charge is made, and students, whether they are matriculants or not, are at liberty to attend. This course is wholly separate from the winter course, which is complete in itself. The dissecting rooms are open, and an opportunity afforded of commencing that important branch of duty. The surgical clinque is in operation each Saturday; the obstetric clinque commences to furnish its cases. Advantage may also be taken of an attendance on the various gratituous institutions in the city, such as the Dispensaries, Eye and Ear Infirmary, &c."

The foregoing is equivalent to a concession that more than a four month's course, of six lectures daily *is* necessary. The faculty state that to pass a month in New York before the lecture term commences, is a "real improvement." If so, why not make it a part of the regular course at once, and render it obligatory? We are naturally led to inquire what becomes of the equivalent for time and money in this preliminary month! But two or three lectures, we believe, are given daily during this period. Still more, what is to preserve the student's morals all this time! Surely, if six lectures a day are subsequently necessary to protect him against city temptations during the regular course, he is in great danger of being absolutely ruined if he follow the reccommendation to pass a month in New-York before lectures commence! We are at a loss to perceive how the two paragraphs we have quoted are to be reconciled. The latter seems to us to involve a concession of the invalidity of the arguments presented in the former.

In making these comments we disclaim any unfriendly feelings toward the Medical Department of the University of the city of New-York. The extension of the lecture terms in Medical Institutions has been reccommended by the American Medical Association, and appears to meet with the general approbation of the profession. We believe the Association and the Profession are right, and we truly regret that the measure does not receive the concurrence of all schools. We are not sure, however, that any Institution, except the University of the city of New-York, has taken decided ground against the measure. Several have already adopted it, and most of those who have not, have signified their willingness to do so, if it is to be generally sustained.

